# Childhood acute lymphoblastic leukemia survival and spatial analysis of socio-environmental risks in Mexico

**DOI:** 10.3389/fonc.2023.1236942

**Published:** 2023-10-12

**Authors:** Jaqueline Calderon-Hernandez, Lizet Jarquin-Yañez, Luis Reyes-Arreguin, Luis A. Diaz-Padilla, Jose Luis Gonzalez-Compean, Pablo Gonzalez-Montalvo, Rebeca Rivera-Gomez, Jairo R. Villanueva-Toledo, Kristal Pech, Oscar Arrieta, Yelda A. Leal

**Affiliations:** ^1^ Facultad de Medicina/CIAAS, Universidad Autónoma de San Luis Potosi, San Luis Potosi, Mexico; ^2^ Global Public Health Program, Boston College, Boston, MA, United States; ^3^ Consejo Nacional de Humanidades, Ciencias y Tecnologías (CONAHCYT), Ciudad de México, Mexico; ^4^ Servicio de Oncología Pediátrica de la Unidad Médica de Alta Especialidad (UMAE), Centro Médico Nacional “Ignacio García Téllez”, Instituto Mexicano de Seguro Social (IMSS), Mérida, Yucatán, Mexico; ^5^ Centro de Investigación y de Estudios Avanzados del Instituto Politécnico Nacional Unidad Tamaulipas, Victoria, Tamaulipas, Mexico; ^6^ Servicio de Oncología Pediátrica, Hospital O’Horán, Servicios de Salud de Yucatán/Facultad de Medicina, Universidad Autónoma de Yucatán, Yucatán, Mexico; ^7^ Facultad de Ciencias de la Salud, Universidad Autónoma de Baja California, Tijuana, Baja California, Mexico; ^8^ Comisión de Salud Fronteriza Mexico-Estados, Registro Poblacional de Cáncer de Tijuana BajaREG, Tijuana, Baja California, Mexico; ^9^ Consejo Nacional de Humanidades, Ciencias y Tecnologías (CONAHCYT) – Fundación IMSS, A.C., Ciudad de México, Mexico; ^10^ Departamento de Epidemiología del Instituto Nacional de Cancerología, Ciudad de México, Mexico; ^11^ Coordinación del Registro Nacional de Cáncer del Instituto Nacional de Cancerología, Ciudad de México, Mexico; ^12^ Registro Poblacional de Cáncer Mérida, Unidad Médica de Alta Especialidad (UMAE), Centro Médico Nacional “Ignacio García Téllez”, Instituto Mexicano de Seguro Social (IMSS), Mérida, Yucatán, Mexico; ^13^ Centro Institucional de Capacitación y Registro de Cáncer, Coordinación de Investigación en Salud, Instituto Mexicano de Seguro Social (IMSS), Ciudad de México, Mexico

**Keywords:** acute lymphoblastic leukemia (ALL), environmental exposure, childhood & adolescent, spatial analysis, cancer registry

## Abstract

**Background:**

Acute lymphoblastic leukemia (ALL) etiology remains largely unknown; incidence patterns by age, sex, and geographical distribution suggest a potential environmental role.

**Aim:**

To identify ALL clusters from four contrasting urban areas of Mexico and to characterize the sources of environmental carcinogens.

**Methods:**

Hospital-based ALL cases (n = 443) diagnosed in children <19 years old from the Metropolitan Zones of Merida and San Luis Potosi, the State of Mexico, and Tijuana were analyzed (2015–2020). ALL cases were coded according to the International Classification of Diseases for Oncology. ALL clusters were identified by Kernel Density, and excess risk was estimated. Data of particulate matter ≤2.5 µm (PM_2.5_) concentrations measured by community-monitoring stations were analyzed. Geocoded datasets of benzene, polycyclic aromatic hydrocarbons, and PM_2.5_ sources were analyzed to characterize patterns of exposure in ALL clusters.

**Results:**

The survival rate for ALL ranged from 61.5% to 78.6%. Seven ALL clusters with excess risk (RR 1.4–2.3, p < 0.05) were identified. The carcinogen sources included artisanal brick kilns, gas stations, cement works, carpentry, paint, and chemical manufacturing establishments. PM_2.5_ levels ranged from 15 µg/m^3^ to 37 µg/m^3^ among study areas.

**Conclusion:**

ALL clusters were identified at the community level; the excess risk could be explained by small-scale carcinogen sources. The levels of PM_2.5_ in outdoor air ranged from 3 to 6 times above the World Health Organization (WHO) air quality guidelines. Healthcare providers must raise awareness of the increased risk of ALL in children living near sources of environmental carcinogens; cancer control and prevention strategies must be steered from a multi-sectoral and multi-action perspective to protect children’s health.

## Introduction

Childhood and adolescent cancer is a significant and relevant public health challenge; each year, an estimated 400,000 children <19 years old are diagnosed with cancer globally. Ninety percent of them live in low- and middle-income countries (LMICs) where treatment is often unavailable or unaffordable. As a result, less than 30% of children with cancer in LMICs survive, compared to more than 80% of those in high-income countries (HICs) (1, 2). The World Health Organization (WHO) Global Initiative on Childhood Cancer proposes reaching at least a 60% cancer survival rate, thereby saving one million lives of children with cancer by 2030 (3). The Global Initiative for Childhood Cancer is part of the response to the World Health Assembly resolution Cancer Prevention and Control through an Integrated Approach, focused on the reduction of premature mortality from non-communicable diseases (NCDs) and the achievement of universal health coverage (4).

Leukemia is the most frequently occurring cancer among children and adolescents; acute lymphoblastic leukemia (ALL) is the most common type, and over 80% of ALL cases are classified as B-lineage (B-ALL). ALL is characterized by the rapid proliferation of poorly differentiated progenitor cells inside the bone marrow (5, 6). Leukemia, among other health conditions, has been included in the group of “new pediatric conditions” (7). Some chemical toxicants in the environment are known and suspected causal factors of the disease (8, 9). It is estimated that 17% (7% to 42%) of all cancer disease burdens in children under five can be attributable to environmental causes; in today’s world, children are surrounded by an estimated 350,000 chemicals (10, 11).

Although the ALL etiology in children and adolescents remains largely unknown, evidence suggests exposure to ionizing radiation, albeit rare, and prenatal/postnatal exposures to pesticides are linked to an increased risk of ALL (12–14). In addition, certain behaviors, such as pica (placing hands and objects in one’s mouth) and playing outside, increase children’s odds of exposure (4). Regarding exposures in urban environments, residential proximity to petrol stations and industrial sites and exposure to mixtures of benzene, particulate matter ≤2.5 µm (PM_2.5_), and polycyclic aromatic hydrocarbons (PAHs) from traffic emission are related to high risk of ALL (15–18).

Interpretation of epidemiologic evidence to determine causality is complex and relies on a wide range of supporting data. The study of cancer and environmental exposures often involves collecting and studying large, complex datasets of cases, environmental exposure patterns or biological indexes, and additional variables (19). In the absence of desirable data on cancer cases from population-based cancer registries (PBCRs), hospital-based cancer registries (HBCRs) could be an alternative.

For this reason, the WHO, through the Global Initiative for Cancer Registry (GICR; www.gicr.iarc.fr) and the International Agency for Research on Cancer (IARC), has promoted establishing cancer registry and surveillance strategies, with standards and quality procedures to provide reliable and better data regarding cancer burden and setting priorities for cancer control and prevention, especially in LMICs.

Therefore, to increase our knowledge and awareness of environmental factors and ALL in Mexican children <19 years old, we designed this research to compare ALL survival rates in four contrasting urban areas (northern, central, and southern). Also, we present an overview analyzing the distribution of the disease from a territorial perspective aiming to identify ALL clusters, visualize areas vulnerable to the disease, and characterize fixed small-scale carcinogens hotbeds at the local/neighborhood level.

## Materials and methods

### Study areas

**Figure 1:**
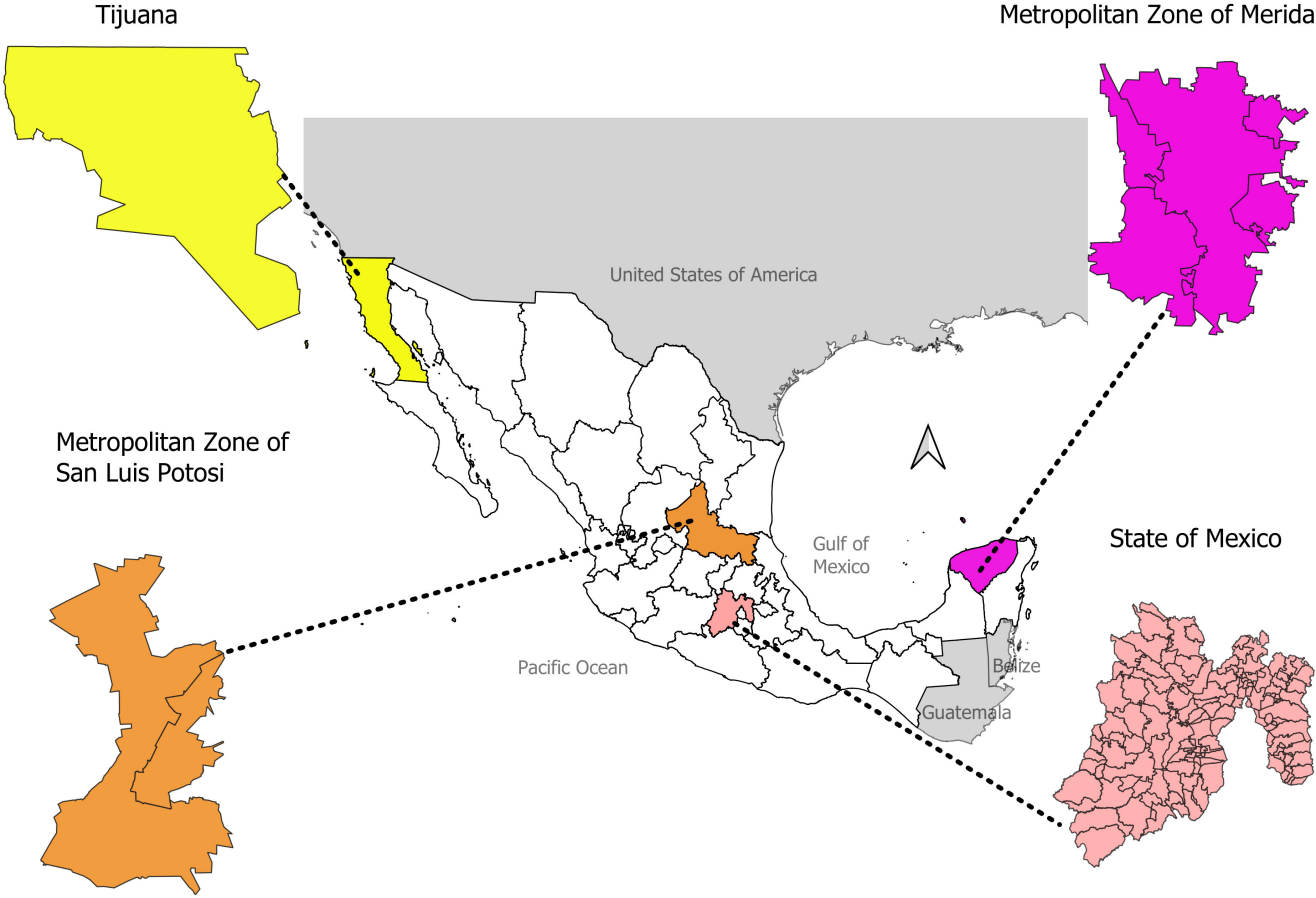
Location of study areas on Mexico geopolitical map.

This study was conducted in four urban areas of Mexico: first, the Metropolitan Zone of Merida (MZ-Merida), located in the southeastern state of Yucatan Mexico (centroid: −89.62166, 20.96777). It includes the municipalities of Conkal, Merida, Kanasin, Ucu, and Uman. Merida City started a PBCR in 2016 to collect data on cancer cases adhering to international standards (20). The MZ-Merida has had accelerated urbanization and economic growth within the last few years; the size of the population is 995,129. Second, the Metropolitan Zone of San Luis Potosi (MZ-SLP) is located in the south-central state of San Luis Potosí (centroid: −100.94282, 22.27046). It includes the San Luis Potosi and Soledad de Graciano Sanchez municipalities, serving 911,908 inhabitants; the area has undergone accelerated industrialization and urban growth within the last decade (21). Third, the State of Mexico (centroid: −99.64537, 19.35596), located in the central part of the country, is a heavily industrialized and densely populated area (1,878,372 inhabitants). It is composed of 125 municipalities, which include Jiquipilco, Otzolotepec, Temoaya, Calimaya, Chapultepec, Lerma, Mexicaltzingo, San Mateo Atenco, Tianguistenco, Toluca, Coatepec Harinas, Ixtapan de la Sal, Tenancingo, and Villa Guerrero. Fourth, the municipality of Tijuana (Tijuana), located in the northwest state of Baja California (centroid: −117.00371, 32.5027), has a population of 922,523. Tijuana borders the southwest region of the United States and is characterized by a high density of gasoline and diesel engine vehicles (22). In 2018, the Tijuana-Cancer Registry was established to monitor and track cancer morbidity in the area (**Figure 1**).

### Source of patient data

Based on hospital records, ALL cases of children <19 years old from the MZ-Merida and Tijuana were included. ALL cases from the MZ-SLP and the State of Mexico were obtained from the registry of the Mexican Association of Aid to Children with Cancer (AMANC by its acronym in Spanish). These are a subset of hospital-based cases. AMANC is a non-profit organization (NPO) involved in the support of the population without medical insurance (formerly Seguro Popular). The HBCR works under international guidelines and standards developed by the IARC and International Association of Cancer Registries (IACR), including the International Classification of Diseases for Oncology (ICD-O3.2) (23, 24). The registry reports pediatric cancer cases through the International Classification of Childhood Cancer (ICCC) (2). Case-finding information was recorded on the following variables: sex, age, date of birth, place of residence, and basis of diagnosis. The HBCR uses CanReg5 software, which prevents the analysis of duplicates and/or non-existent codes. In addition, IARC-Tools and Link Plus 2.0 software were employed as internal quality control measures (25).

The datasets were validated with the IARC-CanReg5 methodology as previously mentioned. Cases with incomplete addresses were excluded to prevent geo-localization. This project was approved by the Health Research Council from the Mexican Institute of Social Security (IMSS by its acronym in Spanish) R-2016-785-065. Case anonymity was maintained by assigning an identifier to each case and eliminating patient names from the dataset.

### Spatial exploratory analysis for ALL cluster identification

A descriptive spatial cluster analysis was calculated by Kernel Density Estimator using QGis^©^. Areas with the highest density of ALL cases were identified according to a distance radius of 2,310 m (meter) for the MZ-Merida, 1,755 m for the MZ-SLP, 12,259 m for the State of Mexico, and 2,215 m for Tijuana. A 200-m cell size was set for each area of study. After ALL spatial clusters were identified, clusters with the highest number of cases were selected, and the area was clipped to obtain a geographic polygon. To estimate the ALL excess risk, a Poisson distribution model was assumed, and the average population of each polygon was obtained from census data (2010 and 2020) belonging to the National Institute of Statistics and Geography (INEGI by its acronym in Spanish). The analysis was conducted with SatScan^©^ software v 10.1.

### Identification of emission sources and geo dataset integration

The latest IARC cancer monographs and National Toxicology Program Reports (US EPA) were consulted, and an exhaustive systematic literature review spanning from 2010 to 2022 was conducted to identify chemical substances associated with an increased risk of ALL in children <19 years old. Benzene, PAHs, and PM_2.5_ (26, 27) were selected to create an inventory of small, fixed sources. A geocoded dataset with the addresses of chemical and paint manufacturing establishments, artisanal brick kilns, gas stations, carpentry, and cement works was created for each area of study. Data were obtained from the National Statistical Directory of Economic Units (DENUE by its acronym in Spanish).

### Scenario characterization by emission sources in the ALL clusters

The polluting emission sources were counted for each Basic Geostatistical Area (BGA) by the vector layer in the UTM Zone 14 N spatial reference system inside each polygon defined by the ALL clusters. Then, hierarchical cluster analysis was carried out to identify BGA clusters for each emission source of pollutants. We used the Euclidean and Manhattan distance matrices and the complete, single, and average linkage among others until the most appropriate number of clusters and the most compact and defined form were obtained. The selected method was the Ward.D2 with the Manhattan distance matrix, and all analyses were conducted with R studio^©^ software (28). The proportion of BGA clusters for emission sources was estimated by each ALL cluster to identify the main emission sources.

### Time trends of annual average measurements of PM_2.5_

PM_2.5_ hourly data for each area of study were obtained from various sources. The MZ-Merida data were acquired from the Mexican National Air Quality Reports (2011–2022). There is a single air quality monitoring station that began gathering data in 2013, but adequate and verifiable data (average/year) were only available from 2015 to 2016 (29). The MZ-SLP data were obtained from the Secretary of Ecology and Environmental Management of San Luis Potosí (30). The MZ-SLP has four monitoring stations; only two reported data for PM_2.5_ for 2016 (30). State of Mexico data were acquired from the National System of Air Quality Information (SINAICA, by its acronym in Spanish) (31), air monitoring started in 2011 (32), and eight stations reported PM_2.5_ (33). Finally, data belonging to Tijuana were also acquired from the Mexican National Air Quality Reports (2011–2022), and only two out of the four monitoring stations in the city reported PM_2.5_ levels (33). It is important to note that only the State of Mexico reported concentrations of PM_2.5_ with enough data (at least 75% of the hourly and daily averages) as required by the Official Mexican Standard (NOM) to analyze time trends (27). Only data that met the criteria of the NOM-025-SSA1-2014 were included in the analysis (29).

### Statistical analysis

Descriptive statistics of ALL cases in CanReg5 were calculated, grouping by age and sex. Survival analysis was performed on R software (version 4.3.0). A non-parametric test was conducted by analyzing Kaplan–Meier curves for ALL cases in the MZ-Merida, the MZ-SLP, the State of Mexico, and Tijuana, and log-rank tests were used to determine the statistical significance of differences. p < 0.05 was considered to indicate statistical significance. Prevalence rates were calculated in each study area by considering average population data from the 2010 and 2020 censuses; age groups under study at the municipal and BGA level were also taken into account (prevalence rates at the BGA level was calculated for the selected ALL cluster polygons). Spatial analysis methods involving Kernel Density Estimation, discrete Poisson modeling, and hierarchical clustering were performed in Rstudio^©^, SatScan^©^, and QGis^©^ software.

## Results

### ALL cases distributed by age, sex, and area of study

This study analyzed a total of 443 ALL cases diagnosed between 2015 and 2020 in children <19 years old. Fifty-nine percent were male (n = 261). Distribution by residence area was as follows: 95 (21.5%) cases from the MZ-Merida, 39 (8.8%) cases from the MZ-SLP, 192 (43.3%) from the State of Mexico, and 117 (26.4%) from Tijuana. Disease distribution among age groups was as follows: 91 cases (20.5%) between 0 and 4 years old, 139 cases (31.4%) between 5 and 9 years old, 119 cases (26.9%) between 10 and 14 years old, and 94 cases (21.2%) for an adolescent group between 15 and 19 years old; data are shown in **Table 1**.

**Table 1:** Demographic characteristics of children <19 years old with acute lymphoblastic leukemia (2015–2020).

	Sex	n	%	Age group (years)	n	%
Metropolitan Zone of Merida	Female	33	34.7	0–4	34	35.8
				5–9	17	17.9
	Male	62	65.3	10–14	30	31.6
				15–19	14	14.7
Metropolitan Zone of San Luis Potosi	Female	16	41.0	0–4	4	10.3
				5–9	17	43.6
	Male	23	59.0	10–14	8	20.5
				15–19	10	25.6
State of Mexico	Female	93	48.4	0–4	15	7.8
				5–9	70	36.4
	Male	99	51.6	10–14	61	31.8
				15–19	46	24.0
Tijuana	Female	40	34.2	0–4	38	32.5
				5–9	35	29.9
	Male	77	65.8	10–14	20	17.1
				15–19	24	20.5

### Survival analysis

**Figure 2:**
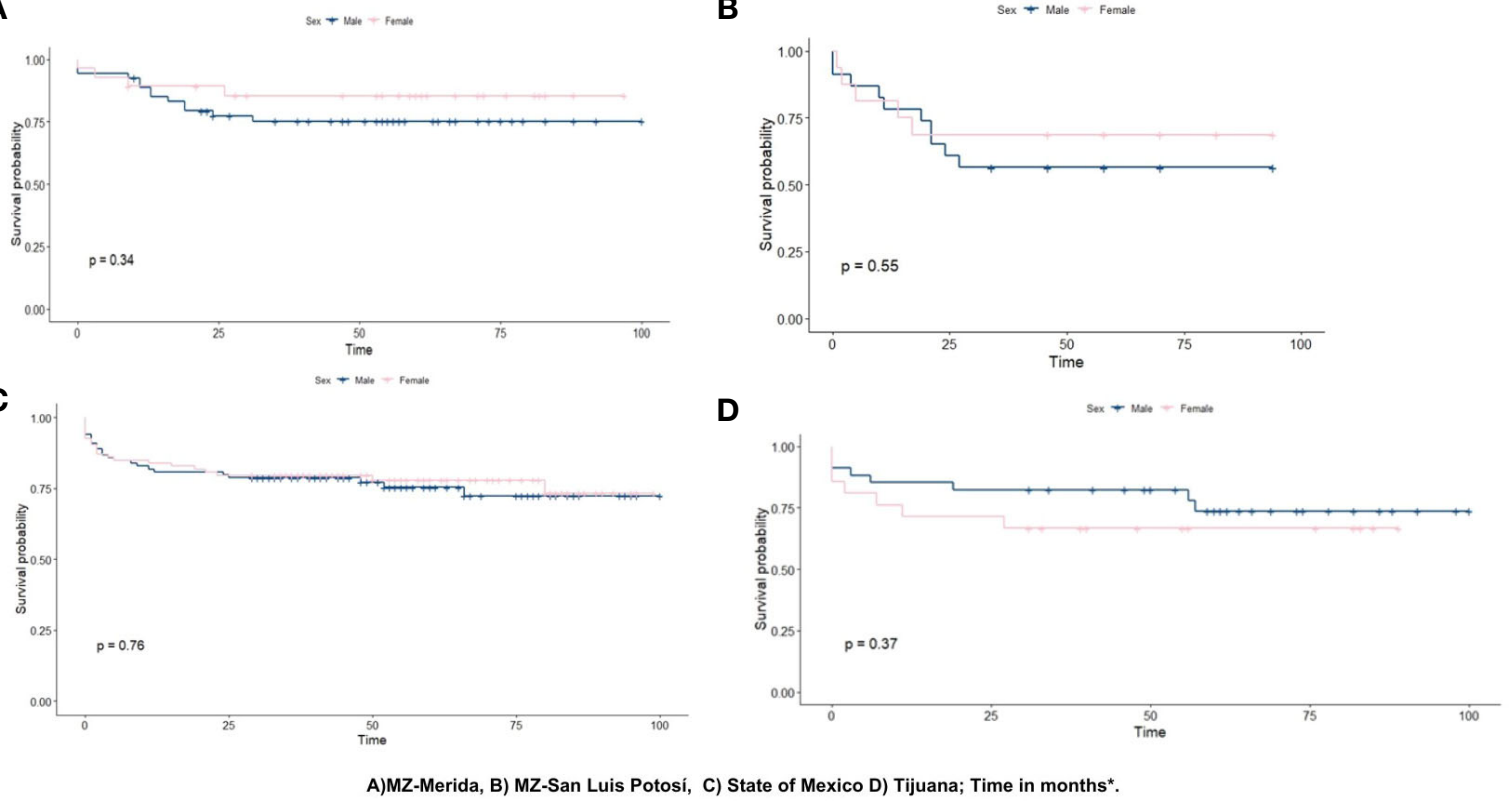
Kaplan–Meier curve for ALL survival in children.

In the MZ-Merida, the 5-year survival rate in children <19 years old diagnosed with ALL was 78.6% (95% CI, 70.1–88.2). Furthermore, survival analysis by sex found that male children in this area had a lower survival rate of 75.3% (95% CI, 64.5–87.9) when compared to female children with a rate of 85.4% (95% CI, 73.1–99.7). These results were not statistically significant (p = 0.34). The MZ-SLP documented the lowest overall survival rate of 61.5% (95% CI, 48.0–78.9). Further analysis by sex found that male children in this area also had the lowest survival rate at 56.5% (95% CI, 39.5–80.9) when compared to female children at 68.8% (95% CI, 49.4–85.7). These results were not statistically significant (p = 0.55). Cases from the State of Mexico had a 72.9% (95% CI, 65.4–81.2) survival rate. Male and female children in this area had a similar survival rate distribution at 72.3% (95% CI, 62.6–83.5) and 73.3% (95% CI, 62.2–86.2), respectively; but these results were not statistically significant (p = 0.76). Finally, Tijuana yielded a 5-year survival rate of 70.6% (95% CI, 58.8–84.8) in children <19 years old. Female children had a 66.7% (95% CI, 49.3–90.2) survival rate, while male children had 73.7% (95% CI, 59.2–91.7), although the difference was not statistically significant (p = 0.37); data are shown in **Figure 2**.

### Spatial ALL case distribution

**Figure 3:**
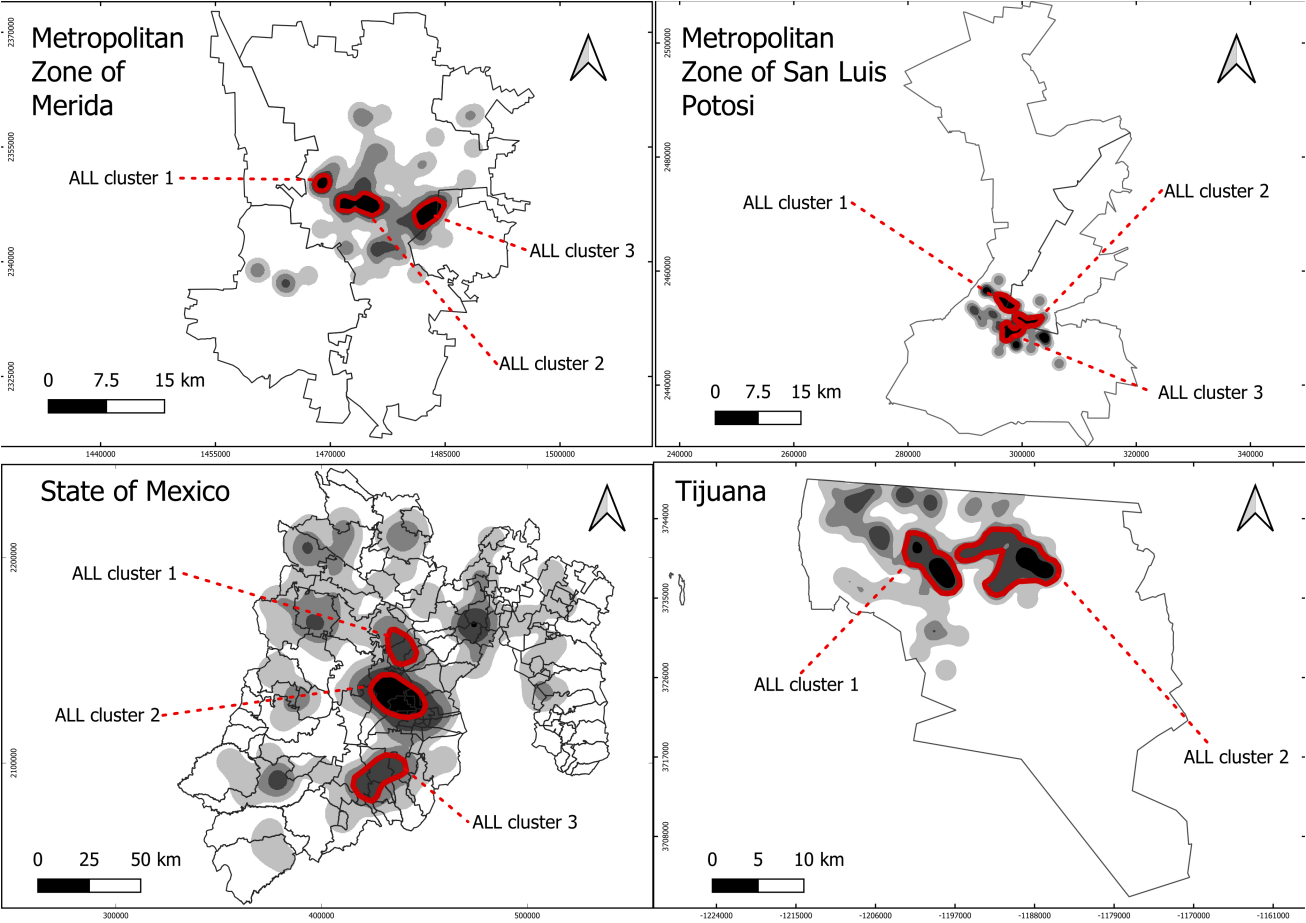
Density of acute lymphoblastic leukemia by study areas. Clusters with the highest number of cases were selected for characterization, identified by a red circle.

The spatial analysis identified 24 ALL clusters distributed by the following: five in the MZ-Merida, six in the MZ-SLP, seven in the State of Mexico, and six in Tijuana. This preliminary spatial analysis selected 11 ALL clusters for carcinogens’ source characterization (identified with a red circle in **Figure 3**): three of them in the MZ-Merida identified as Cluster 1, located in the west-central area; Cluster 2, downtown area; and Cluster 3, located in the central-east area (**Figure 3**). Four cement works, 37 carpentry, 20 gas stations, and three chemical manufacturing establishments were identified in this area.

In the MZ-SLP, three clusters were identified: Cluster 1 in the north-central area, Cluster 2 in the downtown area, and Cluster 3 in the central-south area (**Figure 3**). The analysis geolocated 23 gas stations, 80 brick kilns, 16 paint manufacturing establishments, and six chemical manufacturing establishments in these clusters.

Three clusters were identified within the State of Mexico: Cluster 1 in the north-central region encompassing Jiquipilco, Otzolotepec, and Temoaya municipalities; Cluster 2, in the central region including the Calimaya, Chapultepec, Lerma, Mexicaltzingo, San Mateo Atenco, Tianguistenco, and Toluca municipalities; and Cluster 3 located in the south-central area surrounding the Coatepec Harinas, Ixtapan de la Sal, Tenancingo, and Villa Guerrero municipalities (**Figure 3**). Sources of carcinogens included two cement works, 411 carpentry, 161 gas stations, 230 brick kilns, 12 paint manufacturing establishments, and 50 chemical manufacturing establishments.

In Tijuana, two clusters were identified: Cluster 1 in the west and Cluster 2 in the northeast of the municipality (**Figure 3**). Seven carpentry, 52 gas stations, one brick kiln, three paint manufacturing establishments, and 12 chemical manufacturing establishments were pinpointed.

### Prevalence of ALL and excess risk

The prevalence rate of ALL within the total identified eleven-clusters was 2.4 per 10,000 inhabitants (173 cases within a total population of 709,571). Excess risk (RR) was calculated in each study area by assuming a discrete Poisson distribution. The MZ-Merida had the highest RR at 2.3 (log-likelihood ratio 7.6; p-value 0.00032) in Clusters 1 and 3. The State of Mexico yielded an RR of 2.2 (log-likelihood ratio 5.5; p-value 0.0046), and Tijuana had an RR of 1.4 (log-likelihood ratio 4.1; p-value 0.03). The MZ-SLP yielded a non-significant excess risk (**Table 2**).

**Table 2:** Prevalence and excess of risk for acute lymphoblastic leukemia in selected clusters.

	Cluster	Population at risk^a^	Observed cases	Prevalence rate^b^	Excess risk	p
Metropolitan Zone of Merida	1 (west-central)	10,612	6	5.7	2.3	0.00032
	2 (downtown)	30,984	14	4.5	1.9	0.00032
	3 (East-central)	21,633	12	5.5	2.3	0.00032
Metropolitan Zone of San Luis Potosi	1 (north-central)	31,496	6	1.9	0.8	>0.05
	2 (downtown)	36,430	7	1.9	0.8	>0.05
	3 (South-central)	33,302	7	2.1	0.9	>0.05
State of Mexico	1 (north-central)	17,305	8	4.6	1.9	0.00046
	2 (central)	327,462	39	1.2	0.5	>0.05
	3 (south-central)	27,439	15	5.5	2.2	0.00046
Tijuana	1 (west)	60,067	21	3.5	1.4	0.034
	2 (east)	112,841	38	3.4	1.4	0.034

a Average data from 2010 and 2020 of the National Institute of Statistics and Geography (INEGI).

b Rate calculated per 10,000 inhabitants.

### Hierarchical clustering for identification of main emission sources by BGA

**Figure 4:**
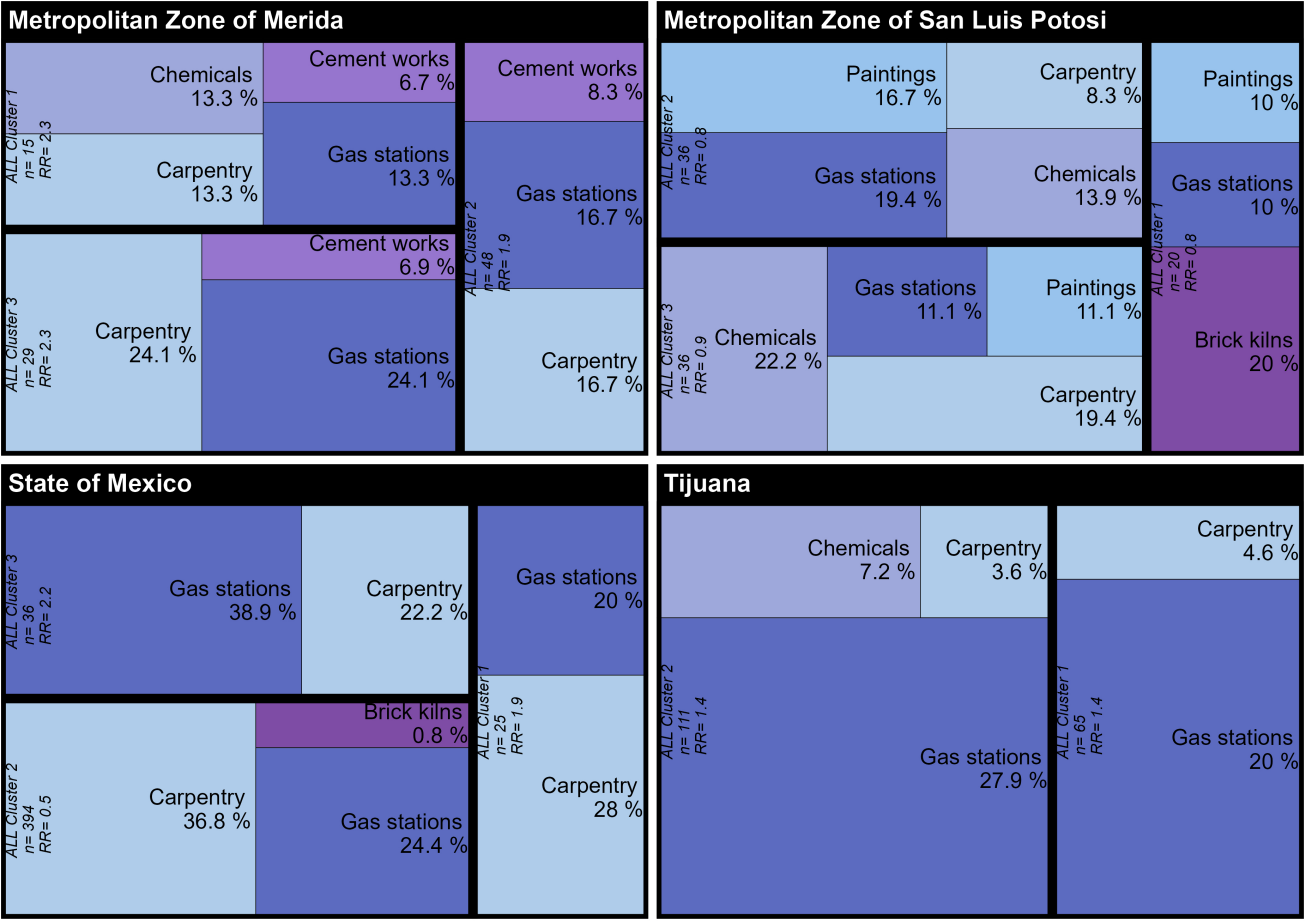
Prevalence of Basic Geostatistical Area (BGA) clusters of fixed polluting sources within the area of the selected ALL (acute lymphoblastic leukemia) clusters.

In the MZ-Merida, the analysis of hierarchical clustering showed a high proportion of BGA clusters of carpentry in the three ALL clusters (13.3%, 16.7%, and 24.1%), gas stations (13.3%, 16.7%, and 24.1%), and cement works (6.7%, 8.3%, and 6.9%) for Cluster 1, Cluster 2, and Cluster 3, respectively. In addition, 13.3% was for chemical manufacturing establishments in Cluster 1. In the MZ-SLP, a high proportion of chemicals manufacturing (22.2%) in Cluster 3, brick kilns (20%) in Cluster 1, and gas stations (19.4%) in Cluster 2 were identified. In addition, painting manufacturing establishments and gas stations were identified in the three clusters. In the State of Mexico, the BGA clusters of carpentry were identified in the three ALL clusters (36.8%, 28%, and 22.2% for Cluster 2, Cluster 1, and Cluster 3, respectively). The BGA cluster for gas stations was 38.9%, 24.4%, and 20% for Cluster 3, Cluster 2, and Cluster 1, respectively. In addition, in Cluster 2, brick kilns were pinpointed. In Tijuana, the high proportion of BGA clusters was gas stations (27.9% and 20.9%) (**Figure 4**).

### Time trends of annual average measurements of PM_2.5_

**Figure 5:**
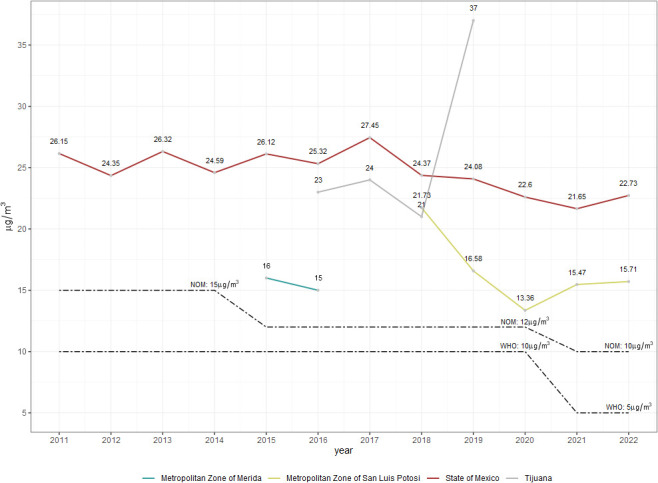
Annual average concentration of PM_2.5_ in the four areas of study in Mexico (2011–2022). Dotted lines represent the World Health Organization (WHO) and the Official Mexican Standard (NOM) air quality guideline changes over time.

The annual average concentrations of PM_2.5_ were analyzed in the four areas of study. The residents in these urban and metropolitan areas were exposed to outdoor air levels of annual average PM_2.5_ that were three to seven times higher than the WHO air quality annual average guideline. In 2019, Tijuana experienced the highest value of PM_2.5_ with 37 µg/m^3^, which was 7.4 times higher than the WHO guideline, while the MZ-Merida had the lowest value (15 µg/m^3^) (**Figure 5**).

## Discussion

The proportion of ALL cases in the four areas of study was higher in male than female; these data are consistent with the report of the global incidence of childhood and adolescent cancer (34). In regard to age, most ALL cases occurred in children aged 0–9 years within the MZ-Merida, the MZ-SLP, and Tijuana. In the State of Mexico, almost 70% of ALL afflicted children aged 5–14 years. This evidence further supports the hypothesis that ALL initiates *in utero*. Molecular proof of prenatal leukemogenesis is provided by backtracking of leukemic somatic mutations (gene translocations) in cord or blood samples at birth (35). Age, area of residence, and paternal or maternal exposures are responsible for differential patterns of exposure to environmental hazards. Exposure to carcinogens during preconception, prenatal, postnatal, and preadolescent periods is critical for ALL onset.

Survival analysis suggests the MZ-SLP had the lowest 5-year survival rate (61.5%) when compared to the national average of 61.8% as reported by a retrospective cohort study (2005–2015) (36). Although the 5-year survival rates for the State of Mexico, the MZ-Merida, and Tijuana (72.6%, 78.6%, and 70.6%, respectively) were higher than the national average, these values fall below those of HICs (37). Differences in survival rates may be attributed to inequities within healthcare system organizations, for example, differences in early diagnosing or early treatment, which are known to increase survival rates. Quality of medical facilities, health insurance, and state of residence are all factors known to further affect disease outcomes. For instance, uninsured children were almost twice to succumb to disease than insured children (34).

The ALL 5-year survival rate within the Mexican population has remained low throughout the years, with a study suggesting it being as low as 58% (38). This survival rate has remained low despite efforts to improve the healthcare system, suggesting additional external or environmental exposures may be at play (39). The analysis shows that children from the MZ-SLP have the lowest survival rate out of all the different areas analyzed, in addition to an extremely high number of known carcinogen sources. One of the clusters analyzed, for example, was composed of 80 artisanal brick kilns (20% of total BGA sources) known to be major mixture carcinogen sources (**Figure 4**). Further research has identified high levels of PAHs, benzene, and epigenetic changes in children living within these neighborhoods (40, 41). A recent publication reported that emissions from brick kilns are especially dangerous sources of pollutants since they employ materials such as oils, tires, wood, and industrial/municipal wastes as combustion materials (42).

ALL cases (ICD-O3.2) reported by HBCRs were analyzed to identify disease clusters containing patterns of small-scale carcinogen sources. Clusters of ALL were identified first, followed by an estimate of excess risk in each area of study. Spatial analysis detected 11 different clusters within the regions analyzed. However, only seven of these clusters had a statistically significant excess risk (RR ranged from 1.4 to 2.3). The highest estimated RR values were found in the MZ-Merida region (**Table 2**). Further analysis of fixed polluting sources in this study area found that 50% of them are related to benzene emissions (gas stations and carpentry), especially in Cluster 1. Chemical manufacturing was also prevalent in this region, accounting for almost 40% of carcinogen sources in Cluster 2 (**Figure 4**).

PM_2.5_ data were only available for the city of Merida from 2015 to 2016, with an annual average of 15 µg/m^3^. This value is three times greater than the WHO’s guideline for outdoor air. In addition to fixed sources of benzene and PAHs, practices such as burning solid wastes, incinerators, and crematories increase levels of carcinogens in the air. Studies have even reported particle-bound PAHs, possibly originating from burning fossil fuels and solid wastes (43).

Drinking contaminated water could be an additional route of carcinogen exposure for children living in the MZ-Merida region since filtration of pollutants by the soil characteristics and karstic aquifer facilitates and degrades drinking water quality in this part of the country (44).

In the State of Mexico, we identified gas stations and carpentry as the major carcinogen contributors (66% according to the type of fixed polluting source identified). The ALL Cluster 3 had an excess risk of 2.2 (p < 0.0046). This cluster comprises the south-central region of the state and includes the Coatepec Harinas, Ixtapan de la Sal, Tenancingo, and Villa Guerrero municipalities, with floriculture being the main economic activity within this area. One of the country’s main flower producers, 60% of the production process in this area is overseen by women, making them and their children vulnerable populations. The flower market has seen strong growth within the past two decades, creating more than 250,000 jobs. The Mexican floricultural market is extremely competitive internationally, with European countries being the main consumers (71% of the world’s production) (45, 46). A highly profitable market, its negative effects on children’s health are often underplayed. Although our research did not focus on pesticide exposure, we identified an area within this region yielding high ALL prevalence. Pesticide exposure is a well-documented, causal factor related to ALL (47). It was very interesting to see a high proportion of gas stations (38.9%) identified within this cluster, in an area where important economic activities demand the transportation of product. Gas stations are a catalyst for carcinogen exposure through gas and diesel combustion. This is a great example of an extremely complex scenario where carcinogen exposure may come from a variety of sources, providing spatial statistical methodologies a critical tool for cancer surveillance.

Disparities in death rates and cancer burdens associated with environmental pollutants are sometimes concealed by aggregated analysis of cancer data at the state, municipal, or medical facility level. In this study, we examined the ALL spatial distribution as a point process to identify highly vulnerable areas for ALL within urban areas. High spatial resolution information on pollution levels and disease rates is necessary to characterize inequities in air pollution, water or economic activities exposures, and related health risks. Hazards inside cities are not distributed evenly, contributing to persistent health disparities between neighborhoods and population sub-groups (48). Thus, spatiotemporal hazard distribution and scenario characterization analysis in small areas must be encouraged to protect children’s health.

Two main clusters were identified in Tijuana, with an ALL excess risk of 1.4 (p < 0.03). Hierarchical cluster analysis demonstrated a significant proportion of carcinogen sources in the form of gas stations (20% and 27.9%). Tijuana is strategically located within the United States–Mexico border and is home to 43 open entry ports. The San Ysidro Port of Entry (POE) is one of the busiest border crossings in the world, overseeing 70,000 vehicles on a daily basis. This area also sees large quantities of industrial imports and exports (e.g., “maquiladoras”), usually transported by large commercial vehicles across the Otay Mesa POE. These two main clusters were located in the northmost region of Tijuana, approximately 5–10 km from the two main POEs. It is estimated that the annual average of PM_2.5_ levels in this area is 7.4 times greater than the WHO air quality guidelines (**Figure 5**).

There is evidence of high benzene concentrations in Tijuana’s air (49). Benzene is the second-highest substance released into Mexico’s air vehicle emissions, with diesel engines being the major contributors (50). Additional sources of benzene include fumes of paint, gas stations, and cigarette smoke. The IARC classified benzene as a human carcinogen, highlighting its morbidity (27). ALL onset through benzene’s toxicological mechanisms in the lung and liver is well understood for cancer in these organs. After inhalation, benzene is metabolized to benzene oxide and hydroquinone. These products are further converted into benzoquinone in bone marrow, initiating cytotoxicity and DNA damage, thereby increasing the risk of developing ALL (51).

Several reports indicate that populations living near industrial or urban areas exhibit a higher proportion of cancer cases, with more than 90% of air pollution-related deaths occurring in LMICs (52). A recent study in Colombia identified leukemia cases clustering within a 1-km radius surrounding industrial sites (53). Another study in San Luis Potosi, Mexico, documented high excess risk in ALL cases clustered near brick kilns, downtown neighborhoods, surrounding industrial zones, heavy traffic zones, and areas with a high density of diesel-burning vehicles (Jarquin-Yañez et al, 2023 data in process of publication). In Spain, García-Perez et al. reported ALL excess risk in children living in urban areas (OR = 1.36; 95% CI 1.02–1.80) (54) and excess risk of ALL in children living within a 2.5-km radius from sources emitting pollutants (OR = 1.31; 95% CI 1.03–1.67) (54, 55). Sources of stationary and mobile polluting sources may include service stations, incinerators, industrial facilities, crematories, urban landfills, artisanal brick kilns, vehicles, and areas burning biomass as fuel (56). These sources emit benzene, PAHs, and PM_2.5_ into the atmosphere. Most recently, these substances have been associated with an elevated risk of pediatric ALL (16, 57, 58). This study was interested in further understanding the geographical distribution of ALL within areas of interest, with the goals of identifying the disease’s spatial patterns and characterizing small-scale carcinogen sources.

Due to the variability in spatial pollutant distribution among cities, cancer surveillance programs should conduct rapid and precise geospatial analysis to identify areas of greatest vulnerability. UNICEF’s 2022 report has urged local, state, and federal governments to prioritize the protection of pediatric environmental health, citing extensive evidence that simultaneous exposure to environmental hazards, even at low doses, can result in adverse health outcomes in exposed populations (59, 60).

The rapid economic and urban growth in urban areas, coupled with weak regulation policies, has increased the likelihood of carcinogen exposure among urban children. International science-based reports of carcinogens now recognize the importance of considering real-world exposure scenarios (61, 62). This approach is especially relevant for countries like Mexico, where 80% of the population resides in urban areas and is exposed to pollutant mixtures on a daily basis (63).

The lack of evidence from epidemiological studies is a critical issue surrounding environmental exposures affecting human health. Most of the evidence comes from studies in countries with solid regulations and bans on pollutants as a control measure. In Mexico, the magnitude of the burden of carcinogens and pollution exposures is poorly documented. Despite well-documented evidence from epidemiological and toxicological studies, pollution is often not recognized as a cause of cancer. Therefore, concrete actions are necessary to translate this evidence into cancer control measures for children and the general population. Children and adolescents who survive cancer are at greater risk of developing other health conditions at early ages (64). Moreover, children are more susceptible to exposure to environmental carcinogens because their organs and systems are developing, and their bodies are smaller compared to adults. In addition, children have specific behaviors such as putting their hands and objects in their mouths and playing outdoors, which increase their exposure to carcinogenic sources (4, 65).

The study has some limitations. The data analyzed were restricted to a period of 5 years (2015 to 2020) for ALL of MZ-SLP and the State of Mexico, and a sub-database of ALL was analyzed. The dataset of sources of exposure was consulted from DENUE, which is a non-exhaustive registry of pollution, and thus, there is a possibility of misclassification bias due to non-registered exposure.

## Conclusion

The results suggest that children <19 years old living in neighborhoods with mixed environmental carcinogenic have an excess risk for ALL. Integrated spatial analysis is necessary to identify vulnerable populations in Mexico. Reducing exposure to carcinogens is essential for cancer prevention. However, it is a challenging task that requires sustained and coordinated actions from national and local governmental authorities. Expanding the network of PBCRs in Mexico is a priority, along with novel surveillance systems that incorporate environmental data and geospatial analysis to identify high-vulnerability areas. Multi-sectoral involvement of the healthcare sector, governmental agencies for environmental monitoring and control, urban planners, researchers, industry, and the population is necessary for cancer control and prevention strategies.

## Data availability statement

The raw data supporting the conclusions of this article will be made available by the authors, without undue reservation.

## Ethics statement

The studies involving humans were approved by Health Research Council from the Mexican Institute of Social Security (IMSS by its acronym in Spanish) R-2016-785-065. The studies were conducted in accordance with the local legislation and institutional requirements. Written informed consent for participation was not required from the participants or the participants’ legal guardians/next of kin in accordance with the national legislation and institutional requirements.

## Author contributions

LY was the lead author of this paper; she conceptualized, wrote, edited, reviewed all sections, and approved the final version for submission. C-HJ conceptualized, wrote, edited, reviewed all sections, and approved the final version for submission. J-YL geocoded the databases and performed the spatial analysis. J-YL, R-AL, PK, V-TJ, and G-CJL reviewed and validated the database, produced the figures and tables, and approved the final version for submission. D-PLA, G-MP, R-GR, and AO edited and reviewed all sections. All authors contributed to the article and approved the submitted version.
